# Rates of Sensorineural Hearing Loss and Revision Surgery After Stapedotomy: A Single-institution Experience Using the Nitinol Prosthesis

**DOI:** 10.1097/ONO.0000000000000025

**Published:** 2022-12-02

**Authors:** Alexandra E. Quimby, Manan Parekh, Nabil F. Darwich, Tiffany P. Hwa, Steven J. Eliades, Jason A. Brant, Douglas C. Bigelow, Michael J. Ruckenstein

**Affiliations:** 1 Department of Otorhinolaryngology—Head & Neck Surgery, University of Pennsylvania, Philadelphia, PA; 2 Lewis Katz School of Medicine, Temple University, Philadelphia, PA; 3 Department of Otolaryngology—Head & Neck Surgery, Temple University, Philadelphia, PA; 4 Department of Head and Neck Surgery & Communication Sciences, Duke University, Durham, NC; 5 Corporal Michael J. Crescenz VA Medical Center, Philadelphia, PA.

**Keywords:** Complications, Otosclerosis, Revision, Stapedotomy, Stapes

## Abstract

**Background:**

Historically, stapedectomy complication rates are quoted as 1% profound postoperative sensorineural hearing loss (SNHL), 5%–10% nonprofound SNHL, and 5%–10% revision surgery.

**Objective:**

We sought to reassess rates of post-stapedotomy complications based on our experience using contemporary surgical technique.

**Methods:**

A retrospective case series was carried out at an academic tertiary referral center. Adult patients undergoing stapedotomy from 2013 to 2020 were included. Primary outcomes were rates of hearing loss and revision surgery. Rates of dizziness, tinnitus, dysgeusia, and proportions of patients who achieved air-bone gap (ABG) closure at 8–12 weeks postoperatively were also assessed.

**Results:**

Four hundred sixty-eight stapedotomies in 399 patients with a median follow-up duration of 99 days (range, 11–5134) were reviewed. One patient (0.21%) suffered profound SHNL and 15 (3.20%) patients suffered nonprofound SNHL. The revision rate for stapedotomies from our institution was 4.49% (21 total revision surgeries). In 277 operations (59.19%), the patient had closure of the ABG within 10 dB. A further 132 (28.21%) had closure of the ABG between 10 and 20 dB. Air pure-tone audiometry scores improved by an average of 25.03 dB. Eighty-three (17.74%) patients complained of postoperative dizziness, which resolved by the time of the first follow-up appointment in all but 26 (5.56%). Seventeen patients (3.63%) complained of tinnitus, and 22 (4.70%) complained of dysgeusia.

**Conclusions:**

SNHL, complications, and revision rates for stapedotomy in the modern era may be substantially lower than those currently presented to patients based on classic techniques and historical data.

Since its first description (1), the procedure of stapedectomy and subsequently stapedotomy has evolved to include a number of surgical techniques (2–5). A variety of prostheses have been introduced; operative lasers have provided a substitute to the drill for creation of fenestrae; and the procedure has been adapted to be performed endoscopically (6–12). Some of these techniques—in particular, the shift from stapedectomy to stapedotomy—have resulted in improved hearing outcomes and operative risk profiles (13–15).

The use of lasers in stapedotomy was first described by Perkins in 1980 (8). Their use has since been adapted to include lasers with different active mediums—primarily, carbon dioxide (CO_2_), potassium-titanyl-phosphate (KTP), and argon—and handheld fiberoptic delivery systems. The modality has become increasingly common over the last 20 years. The laser is used to create a precise, small fenestra in the stapes footplate without attendant mechanical trauma. Doing so may reduce the risk of footplate fracture (16). The laser can additionally be used to focally thin the footplate prior to using a microdrill (or perforator) to create the fenestra, in theory decreasing both mechanical trauma and the spread of thermal energy into the vestibule (17). A recent study demonstrated superior hearing outcomes when comparing the combination of laser (CO_2_ or KTP) and drill to CO_2_ laser or drill alone (18).

With regards to prostheses, a variety of iterations have been introduced over the past decades (6). Teflon, stainless steel, and titanium prostheses have been produced. Prostheses with different means of anchoring to the incus (bucket-handle, wire hook) have become available. Nitinol (titanium and nickel) prostheses—first described in 2005 (7)—are heat-activated to crimp around the incus, and have reduced the risks of too-loose or too-tight manual crimping (which carry attendant risks of delayed failure from incus erosion or necrosis). The results of a 2020 survey administered to the American Otologic Society (AOS) members demonstrated that a majority of respondents favored the use of the Nitinol prosthesis (19).

A potentially devastating complication of stapes procedures is that of profound sensorineural hearing loss (SNHL). While rates of profound SNHL following stapedectomy may be significantly higher, profound SNHL following stapedotomy is frequently quoted as occurring in the range of 1%–2% (20–24). Rates of nonprofound SNHL from stapedotomy have been historically shown to occur in 5%–10% of cases (25–27).

Rates of other benefits and complications commonly cited to patients preparing to undergo stapedotomy are as follows: up to 82% of patients may experience some degree of vertigo, most commonly mild and lasting for up to 48 hours, but a smaller percentage (15% in one series) may have disabling vertigo lasting a week or more (28). Taste disturbance is very common with any manipulation of the chorda tympani nerve (29). Patients with pre-existing tinnitus generally experience improvement or resolution following stapedotomy, but a small percentage (10%) may experience new or worsening tinnitus postoperatively (30). “Success” of stapedotomy is often defined as closure of the air-bone gap (ABG) to within 10 decibels hearing level (dB HL), and the rate in the literature by this definition is up to 94% (20). The need for revision stapedotomy has historically been quoted to patients as occurring in 5%–10% of cases, although several large case series have cited higher rates of up to 20%–30% (31–39).

The impact of contemporary surgical technique on both success and complications of stapedotomy has been less well-studied. Given the number of technological advances in stapes surgery introduced over the last 20 years—in particular, the introduction of the Nitinol prosthesis—we sought to provide an updated understanding of both the success and risks of stapedotomy when performed using these modern techniques.

## METHODS

This study was reviewed by The University of Pennsylvania's Institutional Review Board and received exemption on the basis of being a retrospective chart review (protocol 843656). The electronic medical record (EMR) was queried for all adult patients (age ≥ 18 years) who underwent stapedotomy from 2013 to 2020 at our tertiary academic referral center. This timeframe was selected to ensure that all patients had complete follow-up data; and patients operated on before 2013 were more likely to not have records included in the EMR. All patients operated on during this time period were included, and all procedures were performed by 1 of 4 surgeons. Included procedures were those performed with the indication of otosclerosis. All patients had to have completed at least 1 postoperative follow-up visit, and only patients with postoperative audiograms completed and available for analysis were included. Data were extracted on relevant demographic, clinical history, audiometric, operative, and postoperative variables.

### Outcomes

Our primary outcomes of interest were rates of SNHL and revision surgery. Profound SNHL was defined as postoperative air conduction (AC) hearing thresholds of ≥ 90 dB HL and bone conduction (BC) hearing thresholds worse than the maximum testable level. Nonprofound SNHL was defined as a postoperative BC pure-tone average (PTA) worse than the preoperative BC PTA by ≥ 10 dB HL. Revision rates were defined as the proportion of patients who underwent revision surgery that had undergone their initial stapedotomy procedure at our institution within the past 20 years. We only examined revision rates for patients whose initial surgery was at our institution.

Secondary outcomes of interest were proportions of patients suffering dizziness, tinnitus, dysgeusia, and hearing outcomes based on closure of their ABG to ≤10 or 10–20 dB, as well as the improvements in AC PTAs pre- versus postoperatively. ABGs and AC PTAs were calculated as 3-frequency averages across 500, 1000, and 2000 Hz, and expressed in dB HL.

### Surgical Technique

Variations in technique were employed by the four surgeons, but Nitinol prostheses were used in all cases. For all patients, stapedotomy was performed under general anesthesia with the use of an operating microscope. An endaural incision was made in some cases, based on surgeon preference. A tympanomeatal flap was raised, and removal of the scutum with either a drill or stapes curette and mobilization of the chorda tympani nerve were performed on an as-needed basis to achieve adequate visualization of the stapes footplate, posterior crura, and stapedial tendon. Either a CO_2_ laser fiber (Omniguide) or microscissor was used to release the stapedial tendon and fracture the posterior crura of the stapes, followed by downfracturing and removal of the suprastructure. A fenestra was created in the footplate with either hand perforators (1 surgeon), a combination of CO_2_ laser and microdrill (1 surgeon), microdrill alone (1 surgeon), or CO_2_ laser and hand perforators (2 surgeons); one surgeon used a combination of techniques. A Teflon + Nitinol wire hook prosthesis of appropriate length was placed in all cases, and heat-activated with either the laser or bipolar cautery. Either a single piece of Gelfoam or a blood patch was placed adjacent the prosthesis in the middle ear prior to replacing the tympanomeatal flap. When used, the CO_2_ laser was set at 2 watts of power and 100 ms exposure time.

### Postoperative Course

Patients are discharged the day of surgery. Postoperative follow-up is arranged at approximately 2–3, 8–12 weeks, and then as dictated by the patient’s clinical course.

### Audiograms

All patients underwent preoperative audiometric soundbooth testing. Postoperative audiograms used to determine hearing outcomes were obtained at between 8 and 12 weeks postoperatively. All audiograms were performed according to the following standard protocol. Audiometers are calibrated quarterly according to the International Organization for Standardization requirements. Testing is performed using the Madsen Astera audiometer (Natus Hearing and Balance) and AudBase software (AudSoft Inc). Unmasked BC thresholds are first found using a transducer placed on the mastoid prominence. Calculations of masking requirements are then made automatically and masking is performed using proper narrow band frequencies of appropriate level, with verification of masking by an audiologist when appropriate. AC thresholds are determined with the use of either insert or circumaural headphones. Word recognition scores (WRS) are assessed using a 50-monosyllable word list and reported as percentage correct.

### Statistical Analysis

Descriptive statistics—including means, medians in cases of skewed data, ranges, standard deviations, and interquartile ranges (IQRs)—were used to summarize cohort baseline demographics and surgical outcomes. T-tests and Fisher exact tests were used to assess differences in hearing outcomes and complications in primary versus revision procedures; Bonferroni-adjusted *P* values are reported to account for multiple comparisons. Paired t-tests were used to compare AC PTAs between postoperative and long-term audiograms, for the subset of patients in whom ≥ 12 months follow-up was available. Odds ratios (ORs) and respective 95% confidence intervals (95% CIs) for audiometric outcomes and complications comparing primary and revision procedures, and variations in surgical technique, were calculated using linear and logistic regression, respectively, and Bonferroni-adjusted *P* values are reported. Preoperative hearing levels were adjusted for when calculating ORs of audiometric outcomes. Patients with missing data on dependent or independent variables were excluded from analyses. An alpha value of 0.05 was selected as the cutoff for statistical significance. All statistics were performed using Stata version 15.1 (StataCorp, College Station, TX).

## RESULTS

A total of 520 stapedotomies were performed by 4 surgeons from 2013 to 2020. Fifty-two procedures were excluded due to missing audiometric data, leaving 468 procedures performed in 399 patients. Cohort demographics, including rates of comorbid illness, are summarized in Table 1.

**TABLE 1.** Cohort demographics and surgical technique

	Total = 468 stapedotomies, 399 patients
Age, mean (SD) (years)	49.87 (SD, 12.43)
Female, n (%)	298 (63.68%)
BMI, mean (SD) (kg/m^2^)	27.32 (SD, 5.41)
Right side, n (%)	240 (51.28%)
Revision procedure, n (%)	52 (11.11%)
Family history hearing loss, n (%)	205 (43.99%)
History of head trauma, n (%)	40 (8.55%)
History of congenital hearing loss, n (%)	24 (5.13%)
Comorbidities, n (%)	
Cardiovascular	110 (23.50%)
Renal	10 (2.14%)
Length of follow-up, median (IQR) (days)	99 (IQR, 48–171 days)

BMI indicates body mass index; IQR, interquartile range.

There was 1 case (0.22%) of profound SNHL, with a postoperative AC PTA of 120 dB HL (53 dB HL preoperative AC PTA). 15 patients (3.21%) had nonprofound SNHL, with elevation of their postoperative BC PTA between 10 and 18 dB from its preoperative level (mean 12.6 dB HL).

Fifty-two procedures (11.11%) were revision stapedotomies. However, in 14 of these (26.92%), the original stapedotomy was performed over 20 years prior; an additional 17 were performed within the last 20 years, but at hospitals other than our own. The revision rate for modern stapedotomies, defined as stapedotomies performed at our institution in the last 20 years, was 4.49% (21 total revision procedures), completed at a median of 8 months from the initial surgery (IQR, 4–42 months). In 13 (61.90%) of these revision cases, the patient had no improvement with the initial surgery.

The average preoperative ABG was 33.33 dB HL (SD 10.55). The average postoperative ABG was 11.71 dB HL (SD 8.69). In 277 operations (59.19%), the patient experienced closure of the ABG within 10 dB. A further 132 patients (28.21%) had closure of the ABG between 10 and 20 dB. Among this second group, the median postoperative ABG was 13 dB HL (IQR, 12–15). AC pure-tone audiometry scores improved by an average of 25.03 dB HL after surgery (preoperative mean 58.65 dB HL, range 25–110; versus postoperative mean 33.59 dB HL, range 5–120). One hundred forty-six patients also had an audiogram available at a minimum of at least 12 months’ postoperatively. Among these patients, the average long-term AC PTA was 32.59 dB HL (SD 16.49), indicating hearing stability compared to their immediate postoperative results (mean 33.52 dB HL, SD 14.83) (*P* = 0.230).

Patients experienced postoperative dizziness in 83 cases (17.74%); however, only 26 (5.56%) had persistent dizziness at the time of their first postoperative follow-up visit (approximately 2 weeks after surgery). Postoperative tinnitus was present in 17 cases (3.63%). By the time of last follow-up (median 99 days, IQR 48–171), tinnitus had resolved in 6 of these patients (35.29%), was persistent in 7 patients (41.18%), and persistence versus resolution could not be ascertained in the remaining 4 patients. Dysgeusia was reported in 22 cases (4.70%).

The odds of hearing improvement (closure of the ABG within 20 dB HL or less) was significantly lower in revision versus primary procedures (OR 0.15, 95% CI 0.08–0.28). The odds of postoperative hearing loss, tinnitus, dizziness, and dysgeusia did not differ significantly between primary versus revision procedures (dizziness, OR 1.28, 95% CI 0.63–2.61; dysgeusia, OR 0.37, 95% CI 0.05–2.80; tinnitus, OR 1.07, 95% CI 0.24–4.81). Table 2 summarizes audiometric outcomes and complications, comparing primary and revision procedures.

**TABLE 2.** Complication rates and audiometric outcomes

	All procedures(n = 468)	Primary procedures (n = 416)	Revision procedures (n = 52)	*P*
Preoperative ABG (dB HL), mean (SD)	33.33 (10.55)	33.23 (10.47)	34.09 (11.23)	1.00
Postoperative ABG (dB HL), mean (SD)	11.71 (8.69)	11.01 (8.21)	17.33 (10.33)	<0.0001
Preoperative AC PTA (dB HL), mean (SD)	58.63 (14.86)	58.25 (14.21)	61.78 (18.93)	0.9567
Postoperative AC PTA (dB HL), mean (SD)	33.59 (14.99)	32.30 (14.07)	43.92 (17.96)	<0.0001
Hearing improvement, n (%)	409 (87.40)	378 (90.87)	31 (59.62)	<0.0001
Complications				
SNHL, n (%)	1 (**0.21 %**)	1 (**0.24 %**)	0	1.00
Tinnitus, n (%)	17 (**3.63 %**)	15 (**3.61 %**)	2 (**3.85 %**)	1.00
Dizziness, n (%)	83 (**17.73 %**)	72 (**17.31%**)	11 (**21.15 %**)	1.00
Dysgeusia, n (%)	22 (**4.70 %**)	21 (**5.04 %**)	1 (**1.92 %**)	1.00

Hearing improvement defined as postoperative ABG ≤ 20 dB HL.

ABG, air-bone-gap; AC, air conduction; dB HL, decibels hearing level; PTA, pure-tone average; SNHL, sensorineural hearing loss.

Variations in technique existed both within and between surgeons, particularly in the method used to create footplate fenestra. In 211 cases (46.58%), hand perforators alone were used. A microdrill alone was used in 113 cases (24.94%), and a laser alone was used in 14 cases (3.09%). Combinations of laser and microdrill, or laser and hand perforators, were used in 115 cases (25.39%). Missing operative notes prevented ascertainment of the technique in the remaining 15 cases. There was no significant difference in hearing outcomes or the odds of any post-operative complication or revision across stapedotomy techniques, with the exception of dysgeusia (*P* = 0.024) (Table 3).

**TABLE 3.** Success and complication rates by fenestration technique

Technique	Hand perforators alone (n = 211)	Microdrill alone (n = 113)	Laser alone (n = 14)	Combination (n = 115)	*P*
Hearing Improvement	181 (85.78)	102 (90.27)	12 (85.71)	104 (90.43)	1.00
ABG ≤ 10 dB HL	125 (59.24)	69 (61.06)	10 (71.43)	68 (59.13)	1.00
ABG 10–20 dB HL	56 (26.54)	33 (29.20)	2 (14.29)	36 (31.30)	1.00
Revision, n (%)	27 (**12.80%**)	11 (**9.73%**)	1 (**7.14%**)	9 (**7.83%**)	1.00
SNHL, n (%)	1 (**0.47%**)	0	0	0	1.00
Dizziness, n (%)	33 (**15.64%**)	19 (**16.81%**)	1 (**7.14%**)	28 (**24.35%**)	0.848
Tinnitus, n (%)	7 (**3.32%**)	4 (**3.54%**)	1 (**7.14%**)	4 (**3.48%**)	1.00
Dysgeusia, n (%)	9 (**4.27%**)	1 (**0.88%**)	1 (**7.14%**)	11 (**9.57%**)	0.024

ABG, air-bone gap; dB HL, decibels hearing level; SNHL, sensorineural hearing loss.

## DISCUSSION

As one of the most common otologic procedures, the outcomes and complication rates of stapedotomy have been well-studied. However, given technological advancements over the past several decades, and as surgical technique continues to evolve, there is a need for updated estimates of the risks and benefits of stapedotomy to better counsel patients contemplating surgery. Through analysis of 468 stapedotomy procedures performed between 2013 and 2020, the present study has demonstrated that contemporary stapedotomy likely carries lower risks of SNHL and revision surgery—as well as the other most commonly encountered complications—compared to those historically documented.

The rate of SNHL, both profound and nonprofound, was significantly lower following stapedotomy in our series than traditionally quoted. We found a 0.22% prevalence of profound SNHL, and 3.20% of postoperative nonprofound SNHL. This is in contrast to the oft quoted 1%–2% risk of profound hearing loss following stapedotomy, and 5%–10% risk of nonprofound SNHL. Given that stapedotomies in our series were performed with a variety of techniques to create footplate fenestra, it is difficult to speculate as to an exact cause of our lower observed rates of SNHL compared to other series of stapedotomy in the literature. This heterogeneity lends generalizability to our findings, though, suggesting that an overall low risk of SNHL exists following stapedotomy performed in the modern era.

When considering only primary stapedotomies performed by surgeons at our institution in the last 20 years, we found a 4.49% revision rate. This is comparable to a recent study by Heywood et al (31), which demonstrated a revision rate of 4.5% in stapedotomies performed between 2001 and 2008 using the shape memory Nitinol prosthesis, as was employed in all procedures in our series. These numbers are in contrast to other large, historic case series that have demonstrated higher proportions of patients requiring revision stapedotomy, ranging from 2.6 to 29.4% (20,31–34,36–39). Interpretation of these numbers must be guarded, though, given the risks of: 1) revision surgeries being performed at longer intervals than those captured in our study, and 2) factors such as patient relocation, insurance coverage, or preferences leading them to undergo revision at institutions other than our own. The true rate of revision surgery may therefore be higher than that identified in our series.

Proportions of patients suffering tinnitus (3.63%), dysgeusia (4.70%), and dizziness (17.74% within 0–2 weeks postoperatively, and only 5.56% at 2 weeks postoperatively) were all similarly lower in the present study than those historically estimated.

In the present series, 59.19% of patients experienced closure of their ABG to ≤10 dB HL postoperatively, and 87.39% of patients experience ABG closure to ≤20 dB HL. Among the group of patients in our cohort who achieved ABG closure to 10–20 dB, the median post-operative ABG was 13 dB HL. Our rate of ABG closure to ≤10 dB is lower than the 94% rate previously reported by Vincent et al (35) using a national database containing 2527 patients who had postoperative audiologic data. Their series included patients operated on from 1991 to 2004, with several key variations in the technique: all patients underwent argon laser stapedotomy followed by vein graft interposition, with placement of traditional bucket-handle prostheses. Their patients also had lesser average preoperative ABGs (25.6 dB compared to 33.32 dB HL in our series). Observed differences in our series may therefore be due to 1) differences in underlying patient populations and baseline hearing loss severity, 2) our calculation of 3- rather than 4-frequency PTAs as employed in their study, 3) true differences in outcomes associated with modern surgical technique, and/or 4) other unquantifiable factors. The results reported herein represent an amalgamation of slight variations in technique across four different surgeons, using up-to-date technology, and thus are likely to be widely generalizable to current technique at other institutions. These current results may therefore be a better estimate of the success rates of modern stapedotomy. Notably, a recent study demonstrated similar rates of ABG closure following stapedotomy to ≤10 and ≤20 dB HL of 65.2% and 88.7%, respectively (40).

While all 4 surgeons in our series used the Nitinol prosthesis when performing stapedotomy, variation in the technique otherwise existed on a surgeon-to-surgeon and case-by-case basis predominantly in the method used to create the stapes footplate fenestrae. In a minority of cases (3.09%), a laser alone was used to create the fenestrae. In all other cases, hand perforators alone, microdrill alone, or a combination or laser with either microdrill or perforators were used. Comparing audiometric and other surgical outcomes across techniques, there was no evidence to suggest superiority of one technique over the others. The odds of postoperative dysgeusia was found to differ significantly across techniques; however, this is likely a spurious result that is reflective of other intraoperative factors more directly related to manipulation and preservation of the chorda tympani nerve. A recent survey of AOS members found heterogeneity among surgeons’ preferred techniques in performing stapedotomy—including laser use, prosthesis type, use of a footplate sealant, among other variables—but that a majority (51%) favored the use of the Nitinol prosthesis (19). These variations in technique were also borne out in our cohort across the four surgeons performing stapedotomies at our institution.

That there were 52 patients with missing postoperative audiometric information who were excluded from our study represents a potential source of bias in our risk estimates. If, for example, this missingness is nonrandom but instead indicative of patients with poorer postoperative outcomes failing to pursue audiometric follow-up or seeking care outside of our institution, our estimates of audiometric outcomes and complication rates may be optimistic. Chart review of these 52 patients with missing audiometric data revealed that all but 5 were seen in postoperative follow-up, with none reporting worsened hearing, and 23 who reported improved hearing at first follow-up despite a lack of audiometric data. While this does not eliminate the risk of bias, the most conservative scenario of all patients who failed to present for follow-up having suffered profound SNHL would still make the proportion of patients with profound SNHL following stapedotomy in our series slightly under 1%. Although this represents the most extreme scenario, this risk of bias is inherent to our study design and deserves consideration.

Limitations of the present study include our lack of a historical control group against which to compare audiometric outcomes, rates of complications, and revisions (for which we are limited by the scope of our EMR); our reliance on patient reports and lack of objective data corroborating the presence or absence of postoperative vertigo, tinnitus, and dysgeusia (as well as the presence or absence of tinnitus preoperatively); lack of comprehensive, long-term follow-up on all patients; and a lack of long-term audiometric data on all included patients.

## CONCLUSION

As stapedotomy technique has evolved, so too has there been a need for updated estimates of the rates of success and complications. Through this analysis of 468 stapedotomies performed at our institution using the Nitinol prosthesis, we have provided generalizable estimates of rates of complications, revision surgery, and audiometric outcomes of stapedotomy. We have demonstrated that the proportion of patients who suffer postoperative SNHL and require revision surgery may be much lower than historically estimated; and rates of other common complications are also lower when considering procedures performed using modern surgical technique. This information may be used to better counsel and prepare patients for the benefits and risks of stapedotomy for otosclerosis.

## FUNDING SOURCES

None declared.

## CONFLICT OF INTEREST

None declared.

## DATA AVAILABILITY

The datasets generated during and/or analyzed during the current study are not available.
